# Targeting copper to combat macrophage-driven inflammation: a potential advanced therapeutic strategy

**DOI:** 10.1038/s41392-023-01592-4

**Published:** 2023-09-11

**Authors:** Ralf Weiskirchen

**Affiliations:** Institute of Molecular Pathobiochemistry, Experimental Gene Therapy and Clinical Chemistry (IFMPEGKC), RWTH University Hospital Aachen, Pauwelsstr. 30, D-52074 Aachen, Germany

**Keywords:** Drug discovery, Medical research

In a recent paper published in *Nature*, Solier and coworkers showed that a rationally designed dimer of metformin targeting mitochondrial copper content can effectively oppose macrophage activation and plasticity.^1^ Since macrophages play critical roles in the initiation and maintenance of inflammation, this drug termed supformin holds promise to be a therapeutic effective in suppressing inflammation-related diseases related to macrophages.

Copper (Cu) is an essential trace element acting as a catalyzer in the respiratory chain and being integral part of diverse proteins and metalloenzymes. It further plays important roles in diverse biological functions including growth control, development, connective tissue stabilization, red blood cell formation, and immune regulation.^2^ On the other hand, copper overload models and human diseases associated with excessive copper accumulation such as Wilson’s disease have shown that excess Cu is a potent oxidant causing oxidative stress by formation of reactive oxygen species. That in turn causes severe dysfunction of mitochondrial energy production, molecular and metabolic impairments, and activation of macrophages driving the inflammatory response.^2,3^ However, the exact pathogenesis of inflammation and the contribution of Cu in the activation of macrophages are far from being understood.

**Fig. 1:**
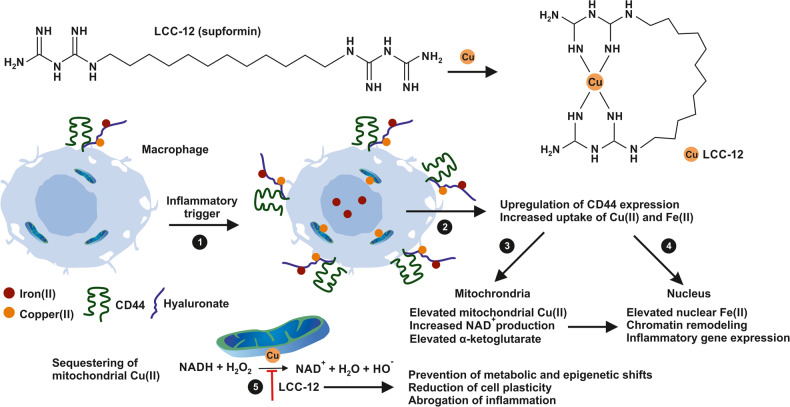
Targeting macrophage-induced inflammation by LCC-12. Macrophage activation is characterized by increased CD44-triggered mitochondrial Cu(II) accumulation and subsequent promotion of NAD(H) cycling. Increased activity of α-ketoglutarate and elevated quantities of nuclear iron trigger metabolic changes and epigenetic modifications leading to inflammatory gene expression. The drug LCC-12 containing two biguanides linked by 12 methylene groups forms specific, highly stable complexes with Cu(II) in mitochondria. The sequestering of Cu(II) by LCC-12 attenuates macrophage activation and dampens inflammatory reactions

Solier and coworkers now identified a pool of chemically reactive Cu(II) in mitochondria of inflammatory monocyte-derived macrophages that is caused by CD44-mediated hyaluronate-bound metal uptake.^1^ The elevated quantities of mitochondrial Cu causes NAD(H) redox cycling through increased expression of superoxide dismutase 2 and decreased activity of catalase. As a consequence the concentration of mitochondrial hydrogen peroxide increases. In the presence of Cu(II) acting as a catalyst NADH reacts with hydrogen peroxide resulting in the formation of NAD^+^ that subsequently initiates metabolic and epigenetic changes leading to macrophage activation and inflammation (Fig. 1). In addition, simultaneous increased iron uptake together with increased quantities of α-ketoglutarate promotes the activity of demethylases that trigger histone demethylation and expression of inflammatory genes. Importantly, the authors now demonstrated that targeting of the mitochondrial Cu(II) pool with a drug termed LCC-12 was effective in preventing the activation and reprogramming of macrophage towards an inflammatory phenotype.

LCC-12 represents a small dimer of metformin composed of two biguanides linked by a stretch of 12 methylene groups. This drug forms highly selective Cu(II) complexes but has no affinity for other divalent metal ions. The in vivo efficacy of this drug in limiting macrophage activation and inflammation was demonstrated in three mouse models of acute inflammation, namely lipopolysaccharide-induced endotoxaemia, cecal ligation and puncture, and a model of viral infection. In all three models that are associated with upregulated CD44 expression and increased cellular Cu, LCC-12 showed highly beneficial therapeutic effects as assessed by reduced expression of markers associated with inflammation, reduction of body temperature, and increased survival rates.

In addition, LCC-12 effectively blocked epithelial-to-mesenchymal transition (EMT) in cancer cells, suggesting that the chelatable mitochondrial Cu pool and its associated signaling pathway have biological functions that go far beyond its involvement in regulating the cellular plasticity of macrophages during inflammatory responses.

The authors further demonstrated that LCC-12 has an about 1000 fold higher activity for Cu(II) than metformin, which is one of the most frequently described hypoglycemic drugs with capacity to interfere with macrophage polarization and reactive oxygen species production. Similar to LCC-12, metformin targets mitochondrial Cu and interferes with EMT in cancer cells.^4^ Thus, LCC-12 is a highly effective super metformin, which makes it understandable why the authors renamed LCC-12 to ‘supformin’.

LCC-12 has a high specificity for Cu(II) that cannot be achieved by other common Cu chelating agent such as ammonium tetrathiomolybdate ((NH_4_)_2_MoS), D-Penicillamine, ethylenediaminetetraacetic acid (EDTA), and trientine hydrochloride (Trien) therapeutically used to remove excess copper. In their study, the authors demonstrated that LCC-12 treatment had no impact on total cellular and mitochondrial Cu content in activated monocyte-derived macrophages, suggesting that LCC-12 does not act as a direct Cu transporter (cuprophore). Nevertheless, sequestering of Cu in mitochondria by LCC-12 might prevent overshooting Cu metalation of mitochondrial enzymes. This could also be of interest in the treatment of other diseases. In Wilson disease elevated mitochondrial Cu deposits cause biophysical, biochemical and electron transport chain deficits in mitochondria, thereby inducing a progressive failure in mitochondrial ATP production.^5^ Consequently, the blocking of detrimental mitochondrial Cu by chelating Cu by LCC-12 might also be beneficial in the management of Wilson disease.

Based on all these findings, chelating Cu by LCC-12 is a conceivable therapy to cure or mitigate inflammatory lesions associated with activation of macrophages. However, it should be mentioned that macrophages are a highly dynamic cell population that not only play critical roles in the induction but also in the resolution of inflammation. Therefore, it will be of fundamental importance to test the efficacy of LCC-12 in models of chronic inflammation. Moreover, the authors showed that LCC-12 also interfered with the activation of dendritic cells and T lymphocytes that similar to macrophages increase CD44 expression upon exposure to specific biochemical stimuli, while not interfering with the activation of neutrophils that is not associated with CD44 upregulation. Therefore, drug carrier and delivery systems designed to selectively target macrophages will be necessary to prevent potential side effects when using this drug in the management of inflammation.

In sum, the fundamental findings of the study of Solier and coworkers might be a significant step forward for the development of anti-inflammatory therapies. The finding that targeting the druggable pool of mitochondrial Cu(II) in activated macrophages by application of LCC-12 allows to interfere with the epigenetic fate of macrophages, the process of EMT, and the progression or outcome of inflammation is of fundamental importance for many research areas. It will now be interesting to follow how these basic research findings will be translated into clinical studies, human applications, and potential new treatments targeting inflammation or other Cu-associated diseases.
